# Increasing breast support is associated with altered knee joint stiffness and contributing knee joint biomechanics during treadmill running

**DOI:** 10.3389/fspor.2023.1113952

**Published:** 2023-04-21

**Authors:** Douglas W. Powell, Hailey B. Fong, Alexis K. Nelson

**Affiliations:** ^1^ Breast Biomechanics Research Center, College of Health Sciences, University of Memphis, Memphis, TN, United States; ^2^ Department of Orthopedic Surgery & Biomedical Engineering, University of Tennessee Health Science Center, Memphis, TN, United States; ^3^ Department of Biomedical Sciences, University of Tennessee Health Science Center, Memphis, TN, United States

**Keywords:** breast, sports bra, knee, biomechanics, running

## Abstract

**Introduction:**

Greater breast support has been associated with improved running performance as measured by oxygen cost and running economy. Several candidate mechanisms have been proposed to underlie breast support-related improvements in running performance including increased knee joint stiffness. Greater knee joint stiffness has been associated with improved running performance (speed and metabolic cost), though the influence of breast support on knee joint stiffness has not been previously investigated. Therefore, the purpose of this study was to investigate the influence of increasing breast support on knee joint stiffness and its constituent components during treadmill running.

**Methods:**

Thirteen recreational runners performed a 3-min running bout at their preferred running velocity in each of three breast support conditions: bare chested (CON), low support (LOW) and high support (HIGH) sports bras. Three-dimensional kinematics and ground reaction forces were collected simultaneously using a 10-camera motion capture system (240 Hz, Qualisys Inc.) and instrumented treadmill (1,200 Hz, Bertec Inc.). Visual3D (C-Motion Inc.) was used to calculate knee joint excursions, moments, powers and work while custom software (MATLAB) was used to calculate knee joint stiffness and breast displacements during the stance phase of running in each experimental condition. A series of 1 × 3 repeated measures analysis of covariance with *post-hoc t*-tests was used to evaluate the effect of breast support on knee joint biomechanics during treadmill running.

**Results:**

Increasing levels of breast support were associated with greater knee joint stiffness (*p* = 0.002) as a result of smaller knee flexion excursions (*p* < 0.001). Increases in knee extension power (*p* = 0.010) were observed with increasing levels of breast support while no differences were observed in knee extension moments (*p* = 0.202) or work (*p* = 0.104).

**Conclusion:**

Greater breast support is associated with increased knee joint stiffness resulting from smaller joint excursions. These findings may provide insight into the biomechanical mechanisms underlying previously reported improvements in running performance including reduced oxygen consumption and greater running economy.

## Introduction

Running is a common form of physical activity with minimal barriers to participation which has been shown to benefit cardiovascular, musculoskeletal and mental health (1–4). While running has many benefits, breast pain is a significant barrier to exercise, including running, for many women with up to 72% of women experiencing exercise-induced breast pain (5–10). Mechanically, exercise-induced breast pain is the result of high tissue strains and strain rates as a function of the passive nature of breast tissue (8, 11, 12). These strain magnitudes and strain rates are evidenced through high breast displacements and velocities. During running, a D-cup breast can experience up to 20 cm of vertical breast displacement and vertical velocities between 80 and 100 cm/s (8, 13, 14). To reduce these tissue strains and strain rates in the passive female breast, external breast support in the form of a sports bra is commonly used. Evidence demonstrates that breast support provided through the use of a sports bra reduces breast displacement in all planes; however, increasing breast support not only influences breast kinematics but has also been shown to alter running kinematics and bioenergetics (9, 15, 16).

Recent research has demonstrated that greater breast support is associated with improved running performance (15). In a sample of female recreational runners, Fong and Powell (15) demonstrated that high compared to low support sports bras were associated with a ∼7% reduction in oxygen consumption and improved running economy while temporospatial characteristics (cadence, step length and ground contact time) were unchanged during a treadmill running task. Moreover, changes in oxygen consumption and running economy were strongly correlated with breast size (*r* = 0.77 and *r* = 0.81, respectively). While Fong and Powell reported changes in running bioenergetics, running biomechanics were not reported (15). Several biomechanical factors were suggested to underlie the observed improvements in running performance including altered transverse plane trunk and pelvis kinematics and increased lower extremity stiffness (15). Evidence has demonstrated that increasing levels of breast support are associated with greater peak trunk and pelvis rotation angles as well as greater trunk and pelvis ranges of motion (9), supporting the assertions by Fong and Powell. The effect of breast support on lower extremity joint stiffness has not been previously investigated.

Knee joint stiffness is a biomechanical measure associated with improved running economy (17). Stiffness is a composite measure that describes a system's response to an applied load (18–21). In running, joint stiffness is characterized by the ratio of the joint moment divided by the joint excursion during the load attenuation portion of the stance phase. Greater knee joint stiffness has been associated with lower oxygen consumption and improved running economy (derived) during treadmill running (17, 22–24). The mechanism underlying stiffness-related changes in running economy is suggested to involve the greater storage and return of energy from the elastic components of the muscle-tendon unit (25).

The biomechanics underlying improved running performance with greater breast support are not well understood. Therefore, the purpose of the current study is to evaluate the influence of breast support on knee joint stiffness during treadmill running. To identify the mechanisms underlying changes in knee joint stiffness, biomechanical factors that influence knee joint stiffness will also be investigating including temporospatial characteristics, knee joint kinetics and knee joint excursions. It was hypothesized that increasing levels of breast support would be associated with greater knee joint stiffness. It was also hypothesized that increasing breast support would be associated with no changes in temporospatial characteristics or knee joint moments, but reduced knee joint excursions. Finally, it was hypothesized that peak knee joint negative powers would be increased while not changes in the negative knee joint work would be observed.

## Methods

An *a priori* power analysis was conducted using knee joint stiffness data from preliminary data. Using an effect size of 0.42 determined from preliminary data, a power (1-*β*) of 0.80 and an *α* of 0.05, a sample size of 12 participants was determined to be an appropriate sample size to detect breast support-related changes in knee joint stiffness values during a treadmill running task. Thirteen participants were recruited as one participant did not complete all three sports bra conditions and was not included in the data analysis. A recreational runner was functionally defined as an individual that ran a minimum of 30 min per day, three or more days per week. Participant inclusion criteria included (1) age 18–35 years, (2) have a self-reported bra size of B-, C-, or DD-cup, and (3) have no history of breast augmentation or reduction surgeries. All participants were required to be free from musculoskeletal injuries for the 6 months prior to data collection. The experimental protocol (PRO-FY2020-24) was approved by the University Institutional Review Board and all participants provided written informed consent prior to study participation (Table 1).

**Table 1:** Participant information including age, height, mass, bust, rib cage, calculated breast size and running speed.

Age (years)	Height (cm)	Mass (kg)	Bust (cm)	Ribcage (cm)	Breast size (cm)	Running speed (m/s)
23.5 (2.8)	1.65 (0.05)	59.8 (4.2)	85.1 (4.3)	75.2 (5.0)	9.9 (4.7)	2.55 (0.21)

Presented as mean (SD).

### Instrumentation

**Figure 1:**
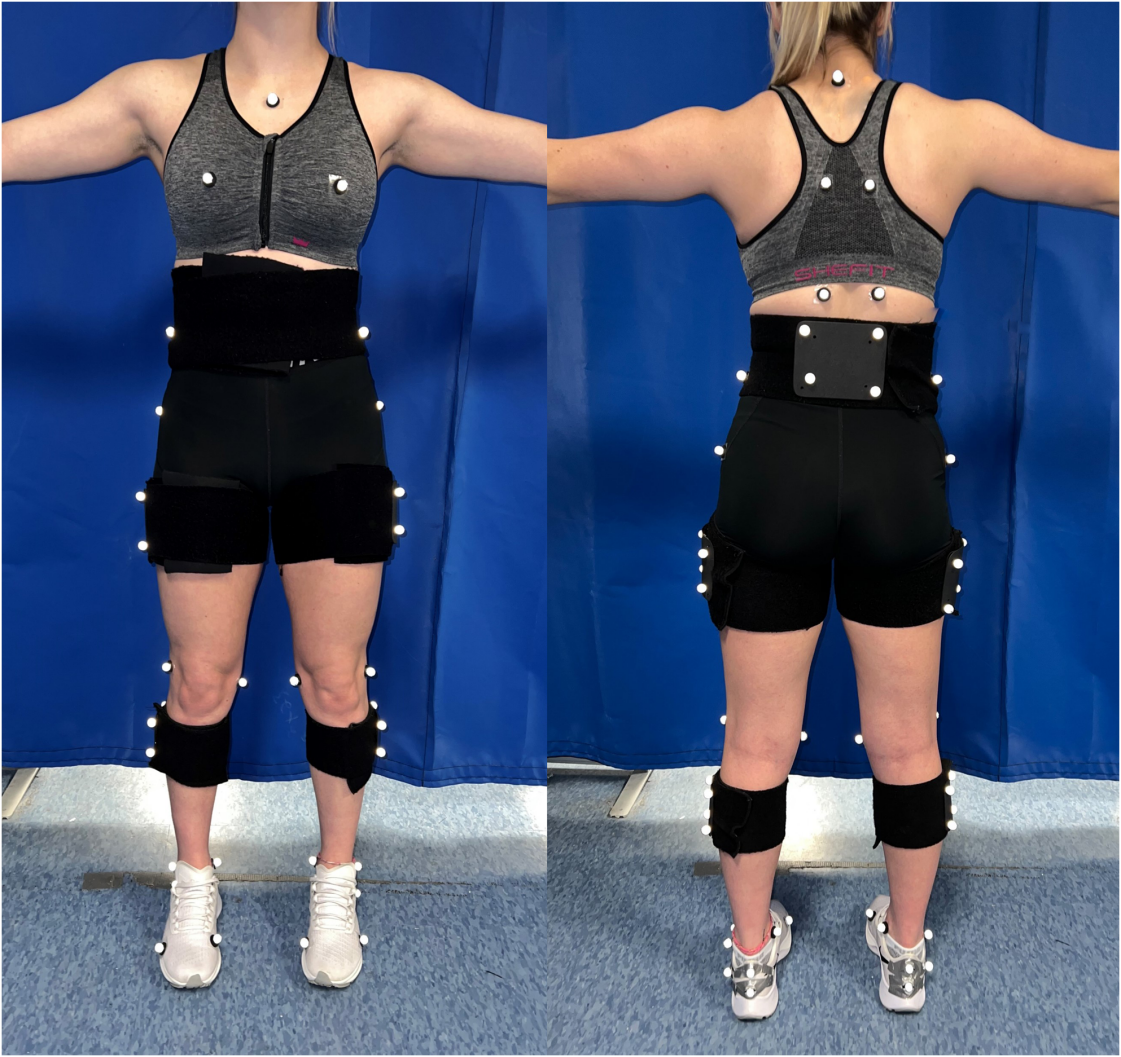
An image depicting the marker set used to evaluate lower extremity biomechanics and breast displacement relative to the torso.

Three-dimensional kinematics and ground reaction forces (GRFs) were recorded using a 10-camera motion capture system (240 Hz, Qualisys AB, Goteburg, Sweden) and an instrumented treadmill (GRFs; 1,200 Hz, Bertec Inc., Columbus, Ohio, United States), respectively. Participants wore spandex shorts and sports bras (dependent on condition) during data collection to limit marker occlusion. Participants completed testing in their personal footwear. The skeleton was modeled using 12.7 mm retroreflective markers and included left and right rearfoot, shank, and thigh segments as well as pelvis and trunk segments (Figure 1). The left and right foot segments were tracked using three individual retroreflective markers placed on the superior, inferior, and lateral calcaneus. The left and right shank and thigh segments, as well as pelvis segment, were tracked using rigid clusters of four retroreflective markers. The trunk segment was tracked using individual retroreflective markers placed over the sternum, and the spinous process of C7, T5 and T12 vertebrae. Anatomical markers for the lower extremities were placed over the right and left first and fifth metatarsal heads, medial and lateral malleoli, medial and lateral femoral epicondyles, greater trochanters, and iliac crests. The trunk was defined using anatomical markers placed over the iliac crests as well as the right and left acromion process (of the scapula). Vertical breast motion was tracked using individual retroreflective markers placed on the manubrium at the sternal notch (sternum) as well as the left and right nipples (10). In the control condition (CON), individual retroreflective markers were placed directly on the skin, while in the low support (LOW) and high support (HIGH) conditions, individual retroreflective markers were placed on the sports bra directly on the nipple. After a standing calibration, all anatomical markers were removed leaving only tracking markers placed on the rearfoot, shanks, thighs, pelvis, trunk and breasts.

### Experimental protocol

Participant anthropometrics were recorded including age (years), height (m), mass (kg), over-bust circumference (cm), and under-bust circumference (rib cage, cm). Bust and ribcage circumference was measured as previously described (26). Participant's breast size was calculated as the difference (in cm) between over-bust circumference and under-bust circumference. Each participant was professionally fitted into two different sports bras: high support (Ultimate, SheFit Inc., Hudsonville, MI, United States) and low support (Flex, SheFit Inc., Hudsonville, MI, United States). Professional fitting was conducted by female research staff who were trained by the manufacturer to properly size and fit the sports bras. The control condition was characterized by the participant performing the experimental protocol bare chested.

Prior to data collection, participants completed a 10-min warmup which included light aerobic activity (stationary cycling) and light dynamic stretching. Following the warmup, each participant performed 3-minute running bouts in each of three randomized breast support conditions (HIGH, LOW, CON). Running velocity was characterized by the participant running on the treadmill at their preferred running pace. To determine preferred running velocity, participants were asked to run across a 20-meter runway at the pace they would select to perform a 5–10 k training run. The participant's running velocity was calculated as the average running velocity of three trials determined using a pair of photocells (63501 IR; Lafayette Instruments Inc., Lafayette, IN) located in the middle of the 20-meter runway. The participant's running velocity was maintained across all running bouts.

Each running bout consisted of 3 min. No data were collected during the first minute to allow the participant to reach metabolic steady state. Two 60-second trials were then collected from the second and third minutes of each running bout. To avoid the confounding factor of fatigue, participants were allowed several minutes of rest between each running bout. At the end of each period of rest, participants provided verbal feedback that they were prepared to continue testing and were not experiencing fatigue. Participants performed a total of 9 min of running over an average period of 60 min of data collection.

### Data analysis

Kinematic and GRF data were filtered using a 4th order, zero-lag Butterworth lowpass filter with cutoff frequencies of 12 and 40 Hz, respectively. These lowpass filter cutoff frequencies were selected as nearly all components of the kinematic and ground reaction force signals of interest exist below these frequencies (27). Lower extremity kinematics, kinetics and GRFs were analyzed from initial contact (IC) to toe off (TO). IC was defined as the instant in which the vertical GRF exceeded 40 N for a period greater than 100 ms; TO was defined as the point after IC in which the vertical GRF fell below 40 N for a period of at least 100 ms. Cadence was calculated as the number of foot strikes (right and left) within each 60-second trial. Stride time was calculated as the difference in time between subsequent IC events. Stride length was then calculated as the product of treadmill velocity and stride time for each stride.

Visual3D (C-Motion, Germantown, MD, United States) was used to calculate lower extremity kinematics and kinetics including sagittal plane knee joint angles, moments and powers. Knee joint excursion was calculated as the difference between knee joint angle at IC and peak knee flexion. Peak knee extensor moment was defined as the maximum value of the knee moment. Knee joint power was calculated as the product of the knee joint moment and the knee angular velocity. Peak negative knee joint power was defined as the minimum value of the knee joint power. Custom software (MATLAB, Natick, MA) was used to calculate negative knee joint work values. Knee joint negative work was calculated as the joint power integrated with respect to time. During the load attenuation phase of running, individual joints oscillate between positive and negative joint powers. The focus of the current investigation pertained to role of the lower extremity during the load attenuation portion of the stance phase of running. As such, only periods of negative joint power were included in the joint work calculation.

The knee joint was modeled as a torsional spring whose stiffness was characterized by the ratio of the change in knee extensor moment relative to the angular displacement of the knee joint during the load attenuation portion of the stance phase of running (19). Custom software (MATLAB, Natick, MA) was used to calculate knee joint stiffness according to Farley et al. (19):

(1) $$k_{knee}=\Delta M/\Delta \theta$$

where *k_knee_* is knee joint stiffness, Δ*M* is the change in joint moment and Δ*θ* is the change in joint angle. Subject means of each dependent variable were used in all statistical analyses and were calculated as the average value across each step of each 60-second running trial.

### Statistical analysis

A Kolmogorov-Smirnov test was used to assess the normality of each dependent variable. All variables were determined to be normally distributed (*p* > 0.05).

As previous research (15) has demonstrated that breast size influences the effect of sports bra support on running performance, a repeated measures analysis of covariance (ANCOVA) was used to evaluate the effect of breast support on running cadence, stride length and contact time, as well as knee joint biomechanics while accounting for the influence of breast size on dependent variables. In the presence of a significant main effect of breast support, *post-hoc* dependent samples *t*-tests were used to compare mean values between breast support conditions. Significance was set at *p* < 0.05 for all comparisons. All statistical analyses were conducted using Prism (GraphPad Inc., San Diego, United States).

## Results

### Vertical breast displacement

**Figure 2:**
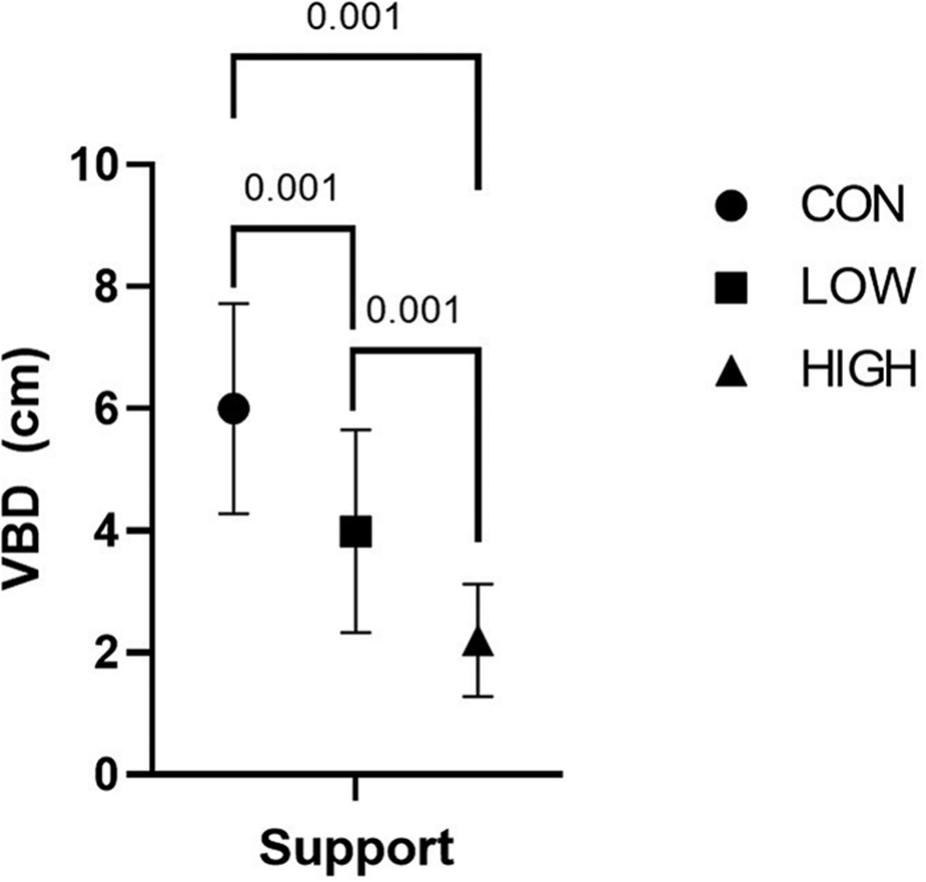
Vertical breast displacement (VBD; in cm) for each of the three breast support conditions: CON, LOW and HIGH.

Relative vertical breast displacement displayed a significant support effect (Figure 2, *F* = 141.56, *p* < 0.001). Tukey's *post hoc* analysis revealed that vertical breast motion was greater in the CON compared to LOW (*p* < 0.001) and HIGH conditions (*p* < 0.001) while the LOW support condition was associated with greater vertical breast motion than the HIGH support condition (*p* < 0.001).

### Temporospatial data

Table 2 presents temporospatial data of running in the CON, LOW and HIGH support conditions. Greater breast support was associated with altered temporospatial characteristics of running including: greater cadence (*F* = 7.18, *p* = 0.002), reduced stride time *F* = 6.70, *p* = 0.003) and reduced stride lengths (*F* = 6.65, *p* = 0.003). Running cadences were slower in CON compared to either the LOW (*p* = 0.009) or HIGH (*p* = 0.002) support conditions while no differences in running cadence were observed between the LOW and HIGH support conditions (*p* = 0.422). Stride time was longer in the CON compared to either LOW (*p* = 0.002) or HIGH support conditions (*p* = 0.010) while no differences were observed in stride time between the LOW and HIGH support conditions (*p* = 0.032). Stride lengths were longer in the CON compared to either LOW (*p* = 0.015) or HIGH (*p* = 0.002) support conditions while no differences in stride length were observed between the LOW and HIGH support conditions (*p* = 0.419).

**Table 2:** Effect of breast support on temporospatial characteristics of running.

Breast support	Cadence (steps/min)	Stride length (m)	Stride time (s)
CON	163.1 ± 8.8	1.89 ± 0.23	0.738 ± 0.041
LOW	164.6 ± 9.7^a^	1.87 ± 0.24^a^	0.731 ± 0.043^a^
HIGH	165.2 ± 8.1^a^	1.86 ± 0.24^a^	0.728 ± 0.037^a^
*p*-value	**0** **.** **002**	**0** **.** **003**	**0** **.** **003**

Presented as mean ± SD.

The bold values represent significant p-values (*p* > 0.05).

^a^ Denotes significant difference compared to CON.

^b^ Denotes significant difference compared to LOW.

### Knee joint stiffness values

Knee joint stiffness values are presented in Table 3. Increasing breast support was associated with greater knee stiffness values (*F* = 7.47, *p* = 0.002). The CON support condition was associated with lower knee joint stiffness values than the LOW (*p* < 0.001) and HIGH (*p* = 0.006) conditions while knee stiffness values were greater in the HIGH compared to LOW conditions (*p* = 0.028).

**Table 3:** Mean kinematic and kinetic variables during the treadmill running task.

Breast support	Stiffness (Nm/kg/deg)	Moment (Nm/kg)	Power (W/kg)	Work (J/kg)	Excursion (deg)
CON	8.4 ± 1.6	2.00 ± 0.33	−8.0 ± 1.9	−0.375 ± 0.096	29.9 ± 5.1
LOW	8.6 ± 1.9^a^	2.00 ± 0.32	−8.1 ± 1.9	−0.376 ± 0.094	29.4 ± 4.9^a^
HIGH	8.8 ± 1.9^a^^,^^b^	2.03 ± 0.35	−8.3 ± 2.0^a^^,^^b^	−0.383 ± 0.093	28.7 ± 5.1^a^^,^^b^
*p*-value	**0** **.** **002**	0.202	**0** **.** **010**	0.104	**<0** **.** **001**

Presented as mean ± SD.

The bold values represent significant p-values (*p* > 0.05).

^a^ Denotes significant difference compared to CON.

^b^ Denotes significant difference compared to LOW.

### Knee joint kinetics

Table 3 presents peak knee extension moments, peak negative knee joint powers and negative knee joint work values during the braking portion of the stance phase. No breast support-related changes in peak knee extension moments were observed (*F* = 1.67, *p* = 0.202).

Increasing levels of breast support were associated with greater negative knee joint powers (*F* = 5.26, *p* = 0.010). While no differences were observed between the CON and LOW support conditions (*p* = 0.305), the CON and LOW support conditions were associated with smaller peak negative knee joint powers than the HIGH support condition (CON-HIGH: *p* = 0.003; LOW-HIGH: *p* = 0.042).

Negative knee joint work was not affected by increasing levels of breast support (*F* = 2.41, *p* = 0.104).

### Knee joint excursions

Knee joint excursions are presented in Table 3. Greater breast support was associated with reduced knee joint excursions (*F* = 16.4, *p* < 0.001). The CON condition was associated with greater knee joint excursions than the LOW (*p* < 0.001) and HIGH (*p* = 0.027) support conditions while the LOW support condition was associated with greater knee joint excursions than the HIGH support condition (*p* < 0.001).

## Discussion

The purpose of the current study was to investigate the effects of increasing levels of breast support on knee joint stiffness and the underlying knee joint biomechanics during running. The major findings of this study demonstrated that greater breast support was associated with greater knee joint stiffness, greater peak negative knee joint powers and smaller knee joint excursions. No differences in peak knee extension moments or knee joint negative work were observed. While previous research has reported that increasing levels of breast support alters running performance (15) as well as trunk and upper extremity kinematics (9, 16) during running, this is the first investigation of the influence of breast support on knee joint biomechanics during a running task.

Greater breast support was associated with increases in knee joint stiffness. Knee joint stiffness is characterized by the ratio of the knee joint extension torque relative to the knee flexion excursion, representing the torsional response of the lower extremity to the applied load associated with the ground reaction force vector (18–21). In running, increases in knee joint stiffness are associated with improved running performance including reduced oxygen consumption and improved running economy when running at a constant velocity (17, 22–24, 28). In the current investigation, increasing levels of breast support were associated with greater knee joint stiffness values. Specifically, compared to the CON condition, the LOW and HIGH support conditions were associated with 2% and 5% increases in knee joint stiffness, respectively, while the HIGH condition exhibited a 2% increase in knee joint stiffness compared to LOW condition. Fong and Powell (15) reported a 7% improvement in running economy when female recreational runners performed a treadmill running task in a high support compared to low support sports bra. Though breast support-related increases in running economy reported by Fong and Powell were greater than the changes in knee joint stiffness observed in the current study, the contribution of knee joint stiffness to improved running economy has not been established. Further, it should be noted that the two studies used different sports bra equipment with the current study using the SheFit Flex (LOW) and SheFit Ultimate (HIGH) sports bras while Fong and Powell had participants wear the Nike Indy (LOW) and Nike Alpha (HIGH) sports bras. It is likely that a disparity exists in the magnitude of breast support provided by the low and high support sports bras used in the two studies of interest.

Reductions in knee joint excursions, not increased knee extension moments, underlie increases knee joint stiffness associated with greater breast support. Knee joint stiffness is calculated as the quotient of the change in knee extension moments divided by the knee joint excursion (change in knee joint angle) during the load attenuation portion of the stance phase of running. In the current study, no changes in knee extension moments were observed with increasing levels of breast support; however, reductions in knee flexion excursion were observed resulting in greater knee joint stiffness. The positive relationship between knee joint stiffness and improved running economy is proposed to be the result of energy storage within and return from the passive, elastic tissues including the parallel and series elastic components of the muscle-tendon unit (24). It is postulated that the reduced knee flexion excursions observed with increasing breast support allow for greater energy storage within the passive, elastic tissues surrounding the ankle and knee joints which are returned during the propulsive portion of the stance phase. Further, this running strategy is selected by the female participants, in part due to the greater control of breast displacement provided by the increasing levels of breast support. In the greater breast support conditions, breast velocities relative to the trunk would be lower in magnitude, reducing the risk of breast pain and injury, allowing the runner to select a metabolically advantageous movement strategy.

Negative joint power and work also provide insight into the breast support-related changes in joint biomechanics. In the current study, increasing levels of breast support were associated with greater knee extension power. Joint power is calculated as the product of the joint moment and joint angular velocity. Given no changes in knee extension moments were observed, it can be assumed that the knee joint angular velocity increased with greater breast support. As the elastic components of the muscle-tendon unit are visco-elastic in nature (29, 30), greater knee flexion velocities would allow greater energy to be stored within and returned from these elastic tissues supporting a reduction in active torque generation and oxygen consumption resulting in a concurrent increase in running economy (17, 23, 24). In the current study, negative joint work was calculated as the negative joint power integrated with respect to time. Therefore, the greater knee extension powers associated with increasing levels of breast support in conjunction with the absence of changes in knee joint negative work suggest that while peak knee extension powers were increased, the duration of power production was reduced indicating a faster movement and supporting the assertion of greater knee flexion velocities.

While the current study presents novel findings regarding the effect of breast support on knee joint biomechanics, some limitations do exist. Though the *a priori* power analysis suggested that a total of 12 participants were necessary for the current study to have sufficient statistical power, the sample size of 12 participants is still small and may not represent the overall population of female recreational runners. Another limitation of the current study pertains to the inclusion of female recreational runners across a spectrum of breast sizes. It is anticipated that running biomechanics of female runners with larger breasts would be more affected by insufficient breast support than female runners with smaller breasts. This is supported by previous research on running economy (15) as well as trunk and upper limb biomechanics (9, 16). The current study did not have a sufficient sample size in each of the various bra sizes to investigate the interaction of breast size and bra support on knee joint biomechanics during running. The interaction of breast size and breast support should be a specific focus of future research studies. A final limitation of this investigation was the use of a preferred running velocity. This running velocity was selected to ensure that participants performed the running task at a comfortable pace that mirrored their common training velocity. However, this suggests that the mechanical demand placed on each female runner during the treadmill running task was not consistent which would limit comparisons between participants. This limitation was addressed through the use of a within-subject design as indicated by the use of a repeated measures ANCOVA. Further, the use of a preferred running velocity represents a more ecologically valid mechanical demand compared to a pre-determined running velocity which may represent a fast or slow running velocity for different participants.

## Conclusion

The results of this study demonstrate that increasing levels of breast support are associated with altered knee joint biomechanics including increased knee joint stiffness, smaller knee joint excursions and greater peak negative knee joint powers. Knee joint stiffness has been associated with both running performance and running-related injury. Therefore, these changes in running biomechanics demonstrate the importance of proper breast support in female runners for both running performance.

## Data Availability

The raw data supporting the conclusions of this article will be made available by the authors, without undue reservation.
